# Vonoprazan-Associated Mucosal Redness: A Report of Two Cases

**DOI:** 10.7759/cureus.71325

**Published:** 2024-10-12

**Authors:** Masaya Iwamuro, Yoshiyasu Kono, Takehiro Tanaka, Seiji Kawano, Nobumasa Ikeda

**Affiliations:** 1 Gastroenterology and Hepatology, Okayama University Graduate School of Medicine, Dentistry, and Pharmaceutical Sciences, Okayama, JPN; 2 Internal Medicine, Clinic Ikeda, Kan'onji, JPN; 3 Pathology, Okayama University Hospital, Okayama, JPN

**Keywords:** endoscopy, gastric mucosal redness, microhemorrhage, potassium-competitive acid blocker, vonoprazan

## Abstract

Vonoprazan, a potassium-competitive acid blocker, is effective at treating acid-related gastrointestinal disorders but has been linked to gastric mucosal redness, a novel condition. This report describes two cases of vonoprazan-associated mucosal redness. Case 1 involved a 73-year-old woman who developed longitudinal erythema and mild mucosal changes after starting vonoprazan seven years ago. Case 2 involved a 70-year-old man who exhibited significant erythema and atrophic gastritis after seven months of treatment. In both cases, the pathological findings included hemorrhage in the superficial mucosa, highlighting that microhemorrhage may be the corresponding pathological finding for vonoprazan-associated mucosal redness.

## Introduction

Vonoprazan, a potassium-competitive acid blocker, has recently emerged as an effective treatment for acid-related gastrointestinal disorders, offering rapid and sustained suppression of gastric acid secretion [1-3]. However, its introduction has been associated with a novel condition of gastric mucosal changes [4-8]. Such gastric mucosal changes include enlargement of fundic gland polyps, enlargement of hyperplastic polyps, multiple white and flat elevated lesions, cobblestone-like mucosa, black spots, white spots, web-like mucus, and mucosal redness [5,6,9,10]. Although vonoprazan has demonstrated efficacy in managing reflux esophagitis and peptic ulcers, the clinical significance of vonoprazan-associated mucosal redness remains poorly understood. This condition, observed during routine endoscopic evaluations, is characterized by erythematous changes in the gastric mucosa that resolve upon discontinuation of the medication. Pathological findings corresponding to this phenomenon have not yet been fully elucidated. This case report aims to provide insights into the endoscopic presentation and pathological findings of vonoprazan-associated mucosal redness.

## Case presentation

Case 1

**Figure 1:**
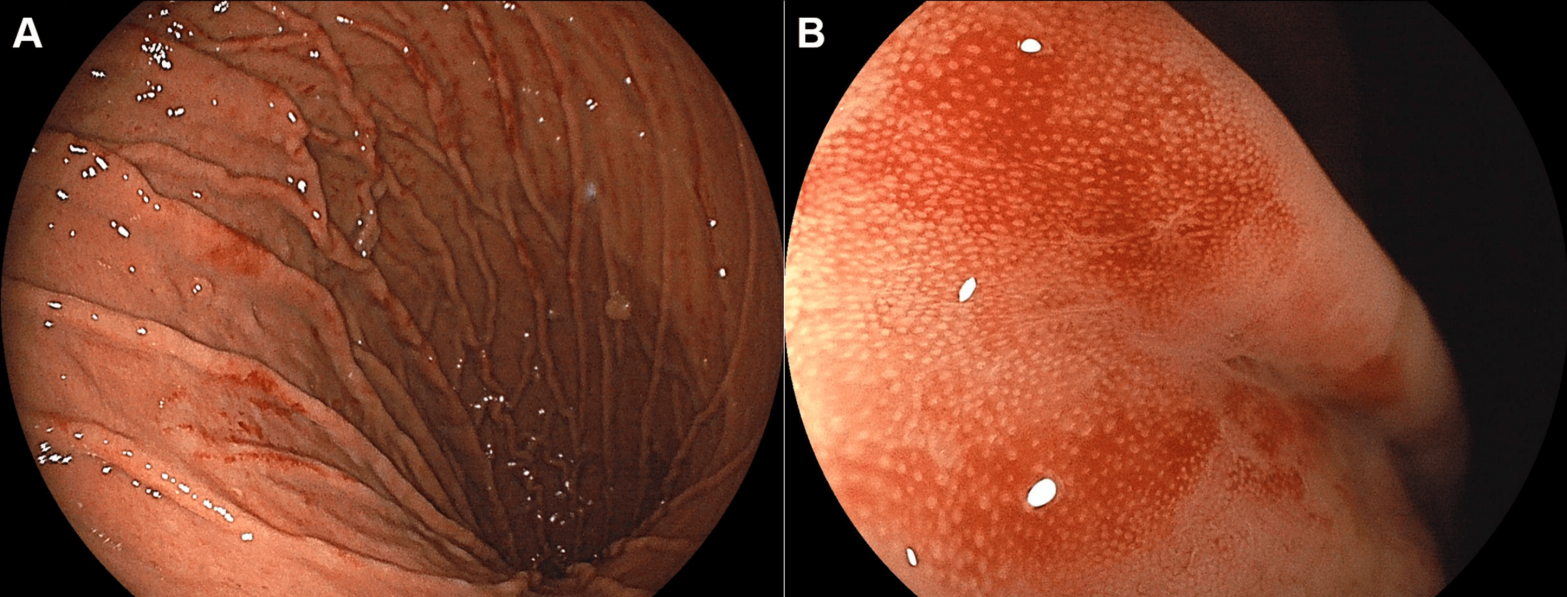
Endoscopic images of case 1. Esophagogastroduodenoscopy shows longitudinal erythema in the gastric body mucosa. A: Endoscopic view of the greater curvature of the gastric body. B: Close-up view of the reddish lesion.

**Figure 2:**
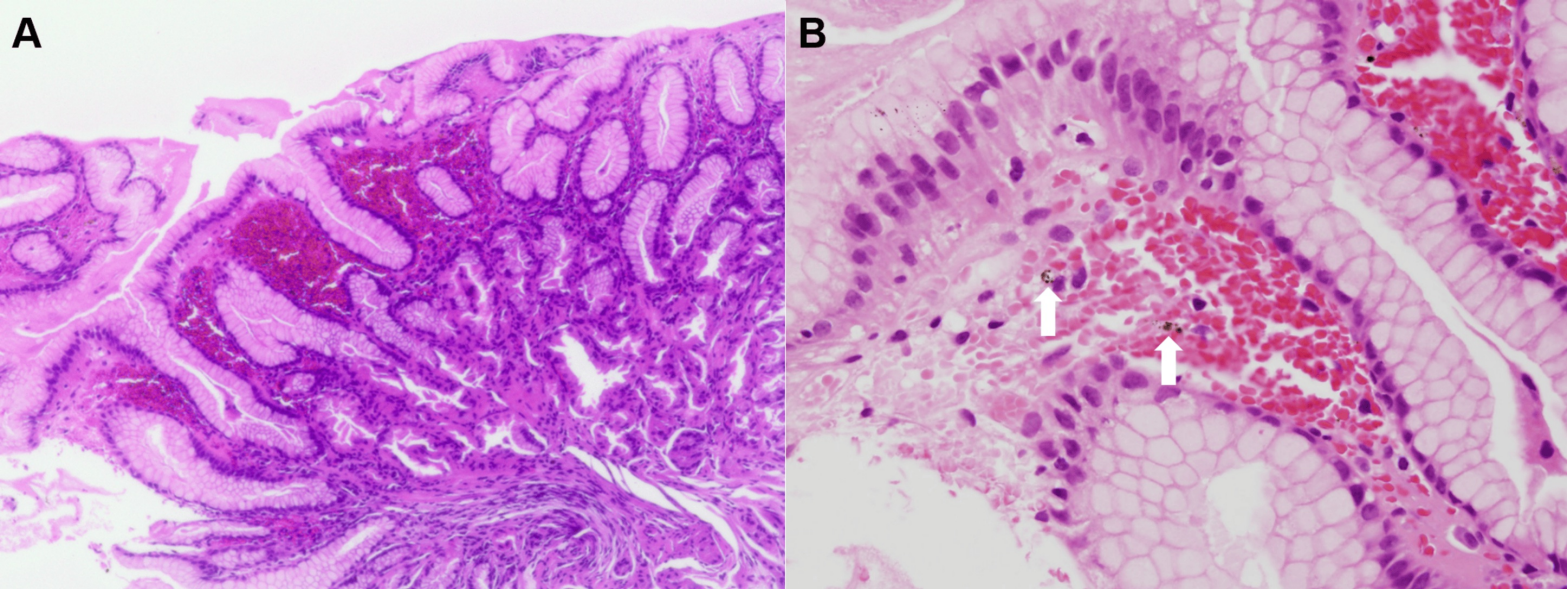
Pathology images of case 1. Hematoxylin and eosin staining of the biopsy specimen reveals extravasation of red blood cells into the superficial mucosa (A: magnification ×5). Higher magnification reveals fine blue-black granular deposits, identified as hemosiderin or hematoidin (B: arrows, magnification ×20).

A 73-year-old Japanese woman underwent esophagogastroduodenoscopy as part of her annual screening. She had been taking rabeprazole and mosapride for reflux esophagitis for seven years, at which point her medication was switched to vonoprazan, which she has continued since then. She also had a history of hypertension, hyperlipidemia, and constipation, for which she was taking amlodipine, simvastatin, and magnesium oxide. The patient was not taking any other over-the-counter medications or supplements. *Helicobacter pylori* infection was ruled out using antibody testing. Esophagogastroduodenoscopy revealed a hiatal hernia, fundic gland polyps, and hyperplastic polyps in the stomach. Longitudinal erythema was observed in the gastric body mucosa (Figure 1). However, no significant differences were observed in the duodenum. A biopsy of the erythematous gastric mucosa revealed mild lymphocytic and plasmacytic infiltration, parietal cell hyperplasia, parietal cell protrusion, vacuolation, and extravasation of red blood cells into the superficial mucosa (Figure 2A). Fine blue-black granular substances were partially deposited and considered to be hemosiderin or hematoidin (Figure 2B, arrows).

Case 2

A 70-year-old Japanese man underwent an esophagogastroduodenoscopy as part of his annual screening. The patient had a history of *H. pylori* eradication eight years prior. He also had comorbidities, including hypertension, diabetes, hyperlipidemia, and arrhythmia, for which he was on long-term medication, including amlodipine, macrogol, metformin, eicosapentaenoic acid, simvastatin, and atenolol. The patient was not taking any other over-the-counter medications or supplements. He underwent endoscopic submucosal dissection for a high-grade adenoma of the duodenum two months ago.

**Figure 3:**
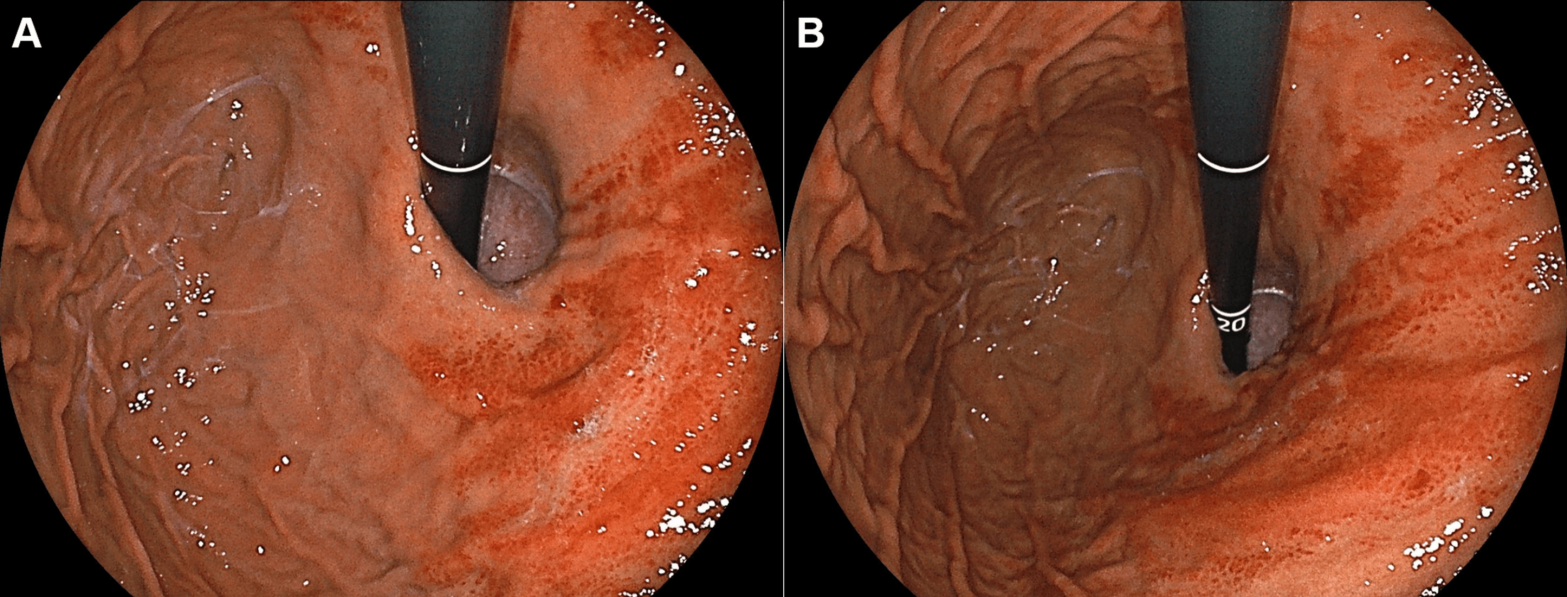
Endoscopic images of case 2 during treatment with vonoprazan. Esophagogastroduodenoscopy reveals significant erythema in the gastric body. A: Endoscopic view of the gastric cardia. B: Endoscopic view of the lesser curvature of the gastric body.

**Figure 4:**
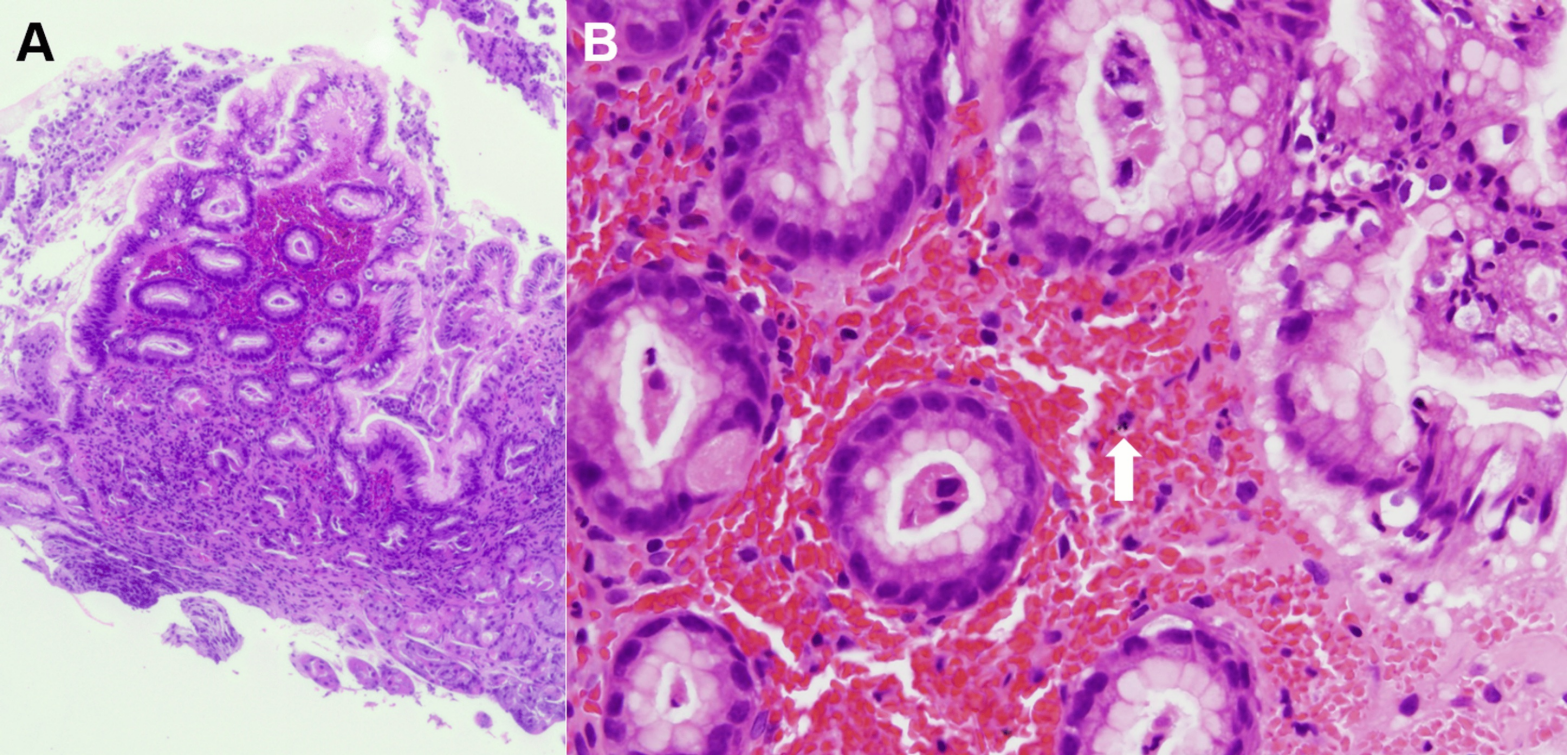
Pathology images of case 2. Biopsy of the erythematous mucosa shows extravasation of red blood cells into the superficial mucosa (A: magnification ×5). Fine blue-black granular substances are partially deposited (B: arrow, magnification ×20).

**Figure 5:**
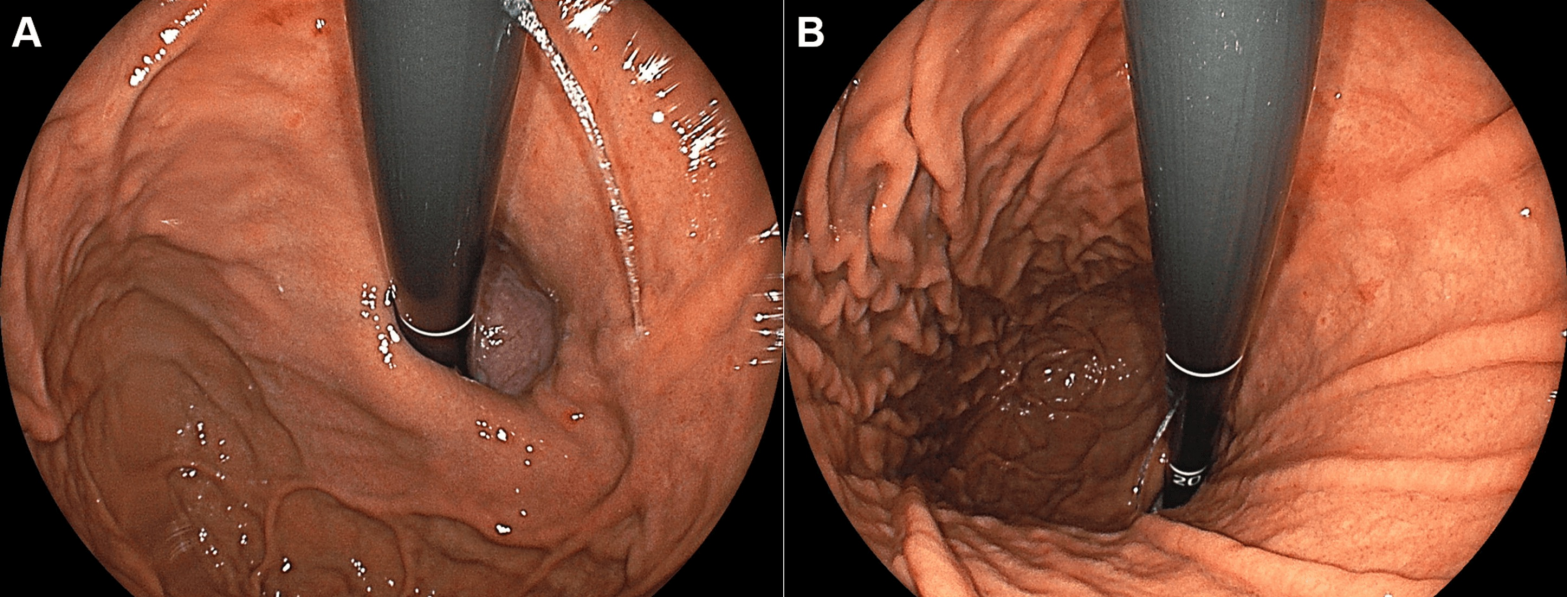
Endoscopic images after switching from vonoprazan to rabeprazole. Resolution of the gastric mucosal erythema is observed. A: Endoscopic view of the gastric cardia. B: Endoscopic view of the lesser curvature of the gastric body.

Seven months prior to esophagogastroduodenoscopy, the patient was diagnosed with Grade B reflux esophagitis and started treatment with vonoprazan. Gastric mucosal erythema was not observed. However, seven months after initiating vonoprazan therapy, esophagogastroduodenoscopy revealed significant erythema in the gastric body (Figure 3), along with atrophic gastritis and hyperplastic polyps. A biopsy of the erythematous mucosa revealed extravasation of red blood cells into the superficial mucosa (Figure 4A). Fine blue-black granular substances were partially deposited and were considered to be hemosiderin or hematoidin (Figure 4B, arrow). The treatment was subsequently changed from vonoprazan to rabeprazole, and a follow-up esophagogastroduodenoscopy one year later showed resolution of the gastric mucosal erythema (Figure 5).

## Discussion

Recently, vonoprazan has been reported to be associated with a distinctive form of gastric mucosal redness [5,6,9,10]. Shinozaki et al. investigated the prevalence of gastric mucosal redness in patients undergoing esophagogastroduodenoscopy [5]. A total of 1,101 patients were included in the study to assess the association between gastric mucosal redness and the use of acid blockers, specifically proton pump inhibitors (PPIs) and vonoprazan. Patients were categorized into four groups: control, H2 receptor antagonist, PPI, and vonoprazan users. Gastric mucosal redness was significantly higher in the PPI (6.2%) and vonoprazan (8.7%) groups than in the control group (1.9%). Multivariate analysis revealed that PPI and vonoprazan use were significantly associated with gastric mucosal redness, with vonoprazan showing a slightly stronger association. This study found no significant relationship between the duration of vonoprazan treatment and the development of gastric mucosal redness, indicating that the condition likely occurs early in therapy. Although the overall prevalence was low, this study emphasized an association between gastric mucosal redness and the use of PPIs or vonoprazan.

The primary mechanism underlying vonoprazan-associated gastric mucosal redness has not yet been determined. Kubo et al. hypothesized that microhemorrhages and congestion were responsible for gastric mucosal redness [9]. Pathological findings from tissue biopsies of the affected areas revealed focal hemorrhage and congestion beneath the basement membrane, accompanied by mild inflammatory cell infiltration, parietal cell protrusions, and oxyntic gland dilatation. Following the discontinuation of vonoprazan, the mucosal redness improved. Subsequent biopsies from the mucosa showed resolution of the microhemorrhage and congestion. The presence of mucosal bleeding in the two encountered cases supports the hypothesis that microhemorrhage is a pathological finding that corresponds to mucosal redness. The presence of fine blue-black granular substances, which were considered hemosiderin or hematoidin (Figures 2B, 4B, arrows), supports the notion that bleeding was not due to biopsy-related artifacts. This is because the formation of hemosiderin and hematoidin takes time and typically indicates a chronic or previous hemorrhage. In contrast, biopsy-related bleeding is often characterized by acute hemorrhage with fresh blood cells. Therefore, the presence of these substances suggests that the hemorrhage occurred prior to biopsy rather than as an artifact of the procedure.

In case 1, the patient was taking amlodipine, simvastatin, and magnesium oxide. In case 2, the patient was taking amlodipine, macrogol, metformin, eicosapentaenoic acid, simvastatin, and atenolol. To date, there have been no reports of these medications causing gastric mucosal redness. However, given that amlodipine was common in both cases alongside vonoprazan, its potential contribution to gastric mucosal redness cannot be excluded. The mechanism underlying the formation of gastric mucosal redness remains unclear, and further investigation is needed to determine whether this condition could be induced by vonoprazan alone or if other medications or comorbidities are involved.

Vonoprazan-associated mucosal redness typically resolves relatively quickly after discontinuation of the medication. Kubo et al. reported that in four cases of vonoprazan-associated mucosal redness, endoscopic findings improved in all cases at three months (three cases) or 12 months (one case) after discontinuation of the drug [10]. The pathological significance of vonoprazan-associated mucosal redness remains unclear; however, diagnosis via endoscopy does not necessarily require discontinuation of the medication. In cases where vonoprazan is not deemed essential, considering a drug holiday or switching to a PPI or H2 receptor antagonist might be an appropriate alternative.

## Conclusions

Vonoprazan-associated mucosal redness is a newly recognized mucosal alteration of the stomach. Although the exact underlying pathophysiological mechanisms by which vonoprazan induces mucosal hemorrhage require further investigation, our cases support the hypothesis that the corresponding pathological finding is a microhemorrhage.
